# MicroRNAs in vitreus humor from patients with ocular diseases

**Published:** 2013-02-20

**Authors:** Marco Ragusa, Rosario Caltabiano, Andrea Russo, Lidia Puzzo, Teresio Avitabile, Antonio Longo, Mario D. Toro, Cinzia Di Pietro, Michele Purrello, Michele Reibaldi

**Affiliations:** 1 Department Gian Filippo Ingrassia, Unità di BioMedicina Molecolare Genomica e dei Sistemi Complessi, Genetica, Biologia Computazionale, Università di Catania, Catania, Italy; 2 Department Gian Filippo Ingrassia, Unità di Anatomia Patologica, Università di Catania, Catania, Italy; 3 Department of Ophthalmology, University of Catania, Catania, Italy

## Abstract

**Purpose:**

Based on evidence that microRNAs (miRNAs) are found in many biologic fluids (e.g., urine, saliva, pleural fluid), we sought to detect the presence of miRNAs and analyze their profile in vitreous humor (VH) from patients affected by various ocular diseases.

**Methods:**

MiRNAs were purified from VH samples taken during vitrectomy, by using the Qiagen miRNeasy Mini Kit. The expression profile on 745 miRNAs was performed by using TaqMan Low Density Array. Single TaqMan expression assays were performed on 18 VH samples (six each from patients with choroidal melanomas, retinal detachment, or macular hole) for miRNAs commonly expressed in serum or retinal cells: let-7b, miR-21, miR-26a, miR-146a, miR-199-3p, miR-210, miR-374a*, miR-532-5p. RNA extracted from serum of six healthy donors or from formalin-fixed, paraffin-embedded samples of choroidal melanocytes from four uveal melanomas (epithelioid cells) and from three unaffected eyes were used as controls.

**Results:**

We identified the presence of 94 circulating small RNAs in the vitreous, some of which (miR-9, miR-9*, miR-125a-3p, miR-184, miR-211, miR-214, miR-302c, miR-452, miR-628, miR-639) are particularly abundant in the VH but downrepresented or not detectable in serum. MiR-146a and miR-26a were overexpressed more than threefold in VH from patients with uveal melanomas compared to the other pathological groups (Wilcoxon signed-rank test, p value <0.05).

**Conclusions:**

Our experimental data suggest that a specific set of circulating miRNAs is secreted in the vitreous, which is quite different from the miRNA pattern in serum, and that the quantity of vitreal miRNAs could change, depending on the pathologies of the eye.

## Introduction

Identification of new diagnostic and prognostic molecular markers for neoplastic and degenerative diseases is a major research area of contemporary medicine. Markers have been characterized for (1) detecting malignant or degenerative diseases early, (2) monitoring their progression, and (3) obtaining reliable information on molecular phenotypes and genotypes to design targeted therapeutic strategies [1,2]. MicroRNAs (miRNAs) are small non-coding RNAs that play a major role as master regulators of protein-coding genes and cell networks. Recent findings indicate that expression of miRNAs is detectably altered in human cancers and that miRNAs may be closely associated with disease development [3]. Altered miRNA expression was reported in various human cancers and other complex diseases [4]. Various types of cancer have distinct miRNA expression profiles, suggesting a specific miRNA signature for each cancer [5,6]. A promising research field on miRNAs has been opened with the identification of miRNAs circulating in the blood and other biologic fluids (e.g., urine, saliva, amniotic fluid, pleural fluid) [7,8]. MiRNAs have been shown to be protected by RNase digestion and are resistant to severe chemical-physical conditions [9]. Accordingly, miRNAs are stable in plasma and serum. These features make miRNAs attractive biomarkers, easily detectable in a non-invasive manner (i.e., blood collection). Patients with different histological tumors have a specific circulating miRNA profile, which is different from that of healthy individuals [10,11]. These data strongly suggest that the use of miRNAs as tumor markers (diagnostic or prognostic) represents a promising field of research. MiRNAs have been identified in many ocular tissues and have been shown to play a role in lens and retina development [12-14], ocular physiology [15], and several ocular diseases. Differential miRNAs expression was found in transparent and cataractous lens [16]. Let-7b is overexpressed in lens with greater opacity [17]; miR-29b is downregulated in Tenon’s fibroblasts after glaucoma surgery, increasing the risk of fibrosis [18]; and miR-24 causes inactivation of p53 surveillance, contributing to tumor development in retinoblastoma [19]. Two miRNAs have been shown to be downregulated in retinal endothelial cells from diabetes patients: MiR-146a-reduced expression is related to an increase in extracellular matrix fibronectin production [20], while miR-200b downregulation is involved in vascular endothelial growth factor (VEGF) alterations in diabetic retinopathy [21]. Overexpression of several miRNAs has been reported in ischemia-induced retinal neovascularization [22]. MiRNAs have also been studied in uveal melanocytes and uveal melanoma cell lines. MiR-137 acts as a tumor suppressor in uveal melanoma cell proliferation through downregulation of its targets *MITF* and *CDK6* [23]; miR-34a acts as a tumor suppressor in uveal melanoma cell proliferation and migration through downregulation of *c-Met* [24]. Since currently no data are available about the presence of miRNAs in human vitreous humor, the aim of this study was to investigate their presence in patients with different ocular diseases and relative controls.

## Methods

This is a comparative study performed at the University of Catania, Catania, Italy during the past 2 years (up to March 31, 2012). Our research followed the tenets of the Declaration of Helsinki; informed consent was obtained from the subjects after explanation of the nature and possible consequences of the study. The VH samples were taken from 18 patients that underwent vitrectomy at the Eye Clinic of the University of Catania: six samples were from patients with uveal melanoma (4 males and 2 females, mean age 62±7 years; Group A), six from patients with retinal detachment (3 males and 3 females, mean age 64±6 years; Group B), and six were from patients affected by macular hole (3 males and 3 females, mean age 59±6 years; Group C). Only patients aged between 50 and 70 years were included. We excluded from our study patients with systemic disease (such as diabetes, renal or hepatic failure, autoimmune diseases, Parkinson and Alzheimer disease), other ocular diseases (such as uveitis, glaucoma, diabetic retinopathy and other retinopathies), previous ocular surgical procedures (including cataract surgery) and trauma, and metastatic disease for patients affected by uveal melanoma. Since radiotherapy could cause optic neuropathy, uveal melanomas extended close or up to the eye disc margin were treated with endoresection [25]. All patients included in Group B were affected by rhegmatogenous retinal detachment with no proliferative vitreoretinopathy, no posterior breaks, and giant retinal tears. Group C included patients with unilateral idiopathic macular hole Gass stages 3 and 4. All vitrectomies were performed by a vitreoretinal surgeon. The infusion port, light pipe, and vitreous cutter were inserted 4 mm from the limbus. The conjunctiva and sclera were opened, and the vitreous cutter was advanced across the vitreous cavity. A 2-ml vitreous specimen with closed infusion was aspirated into a syringe using a three-way tap. The aspirate was then placed in a sterile container, and surgery continued routinely. Samples were centrifuged at 700 ×g for 10' to pellet and remove any circulating cell or debris. RNA was extracted from 500-μl humor vitreous samples by using a Qiagen miRNeasy Mini Kit (Qiagen, GmbH, Hilden, Germany), according to the Qiagen Supplementary Protocol for purification of small RNAs from serum and plasma, and finally eluted in a 30-μl volume of elution buffer. RNAs were quantified by fluorometer and spectrophotometer.

To profile the transcriptome of 745 miRNAs, 4.5 μl of vitreal and serum RNAs (corresponding to 10 ng of RNA) were retrotranscribed and preamplified. Amplified products were loaded on TaqMan Low Density Arrays (TLDAs) TaqMan Human MicroRNA Array v3.0 A and B (Applied Biosystems, Foster City, CA). PCRs on TLDAs were performed on a 7900HT Fast Real Time PCR System (Applied Biosystems). These experiments were performed on vitreous and serum from three patients affected by macular hole. The same amount of vitreal RNAs was used for miRNA-specific reverse transcription (RT) to obtain miRNA-specific cDNAs. Four-fifths of the cDNA total volumes were analyzed with quantitative real-time polymerase chain reaction (RT–PCR) using TaqMan MicroRNA Assays (Applied Biosystems). We assayed the presence of eight miRNAs (let-7b, miR-21, miR-26a, miR-146a, miR-199-3p, miR-210, miR-374a*, miR-532-5p), which were identified at different expression levels through TLDAs and are known to be commonly expressed in serum or in retina cells (the cells in direct contact with VH) [26-29]. To validate our results, the experiments were independently repeated three times, and the standard deviation of threshold cycle (Ct) for each RT–PCR experiment was calculated. We used serum RNAs from six healthy donors as controls. They were selected from hospital personnel, with the same exclusion criteria previously cited, and the serum RNAs were extracted and quantified with the same procedures. We also used RNAs from formalin-fixed, paraffin-embedded (FFPE) samples of normal retinal cells from three unaffected eyes and samples of four uveal melanomas (epithelioid cells), extracted as reported by Ragusa et al. [30]

### Statistical analysis

Since normalizator genes are unknown for VH, we applied the canonical comparative cycle threshold (ΔΔCt) method to perform relative quantification, by using the median Ct of each sample as the normalizator gene and serum as the calibrator sample [30,31].

Differentially expressed miRNAs between vitreous and serum analyzed with TLDAs were identified by SAM (Significance Analysis of Microarrays), applying a two-class paired test among ΔCt and using a p value based on 100 permutations; imputation engine: K-nearest neighbors (10 neighbors); False Discovery rate <0.15.

The Wilcoxon signed-rank test (p value <0.05) was applied to statistically evaluate the expression differences between vitreous and serum and among classes of ocular diseases, analyzed with single TaqMan assays. The partition in expression groups from vitreous was made by calculating the mean Ct value and assuming that the normally expressed miRNAs had a Ct in the range of mean Ct±standard deviation, highly expressed miRNAs had a Ct value less than mean Ct – standard deviation, and lowly expressed miRNAs had a Ct value major than mean Ct+standard deviation.

## Results

**Figure 1:**
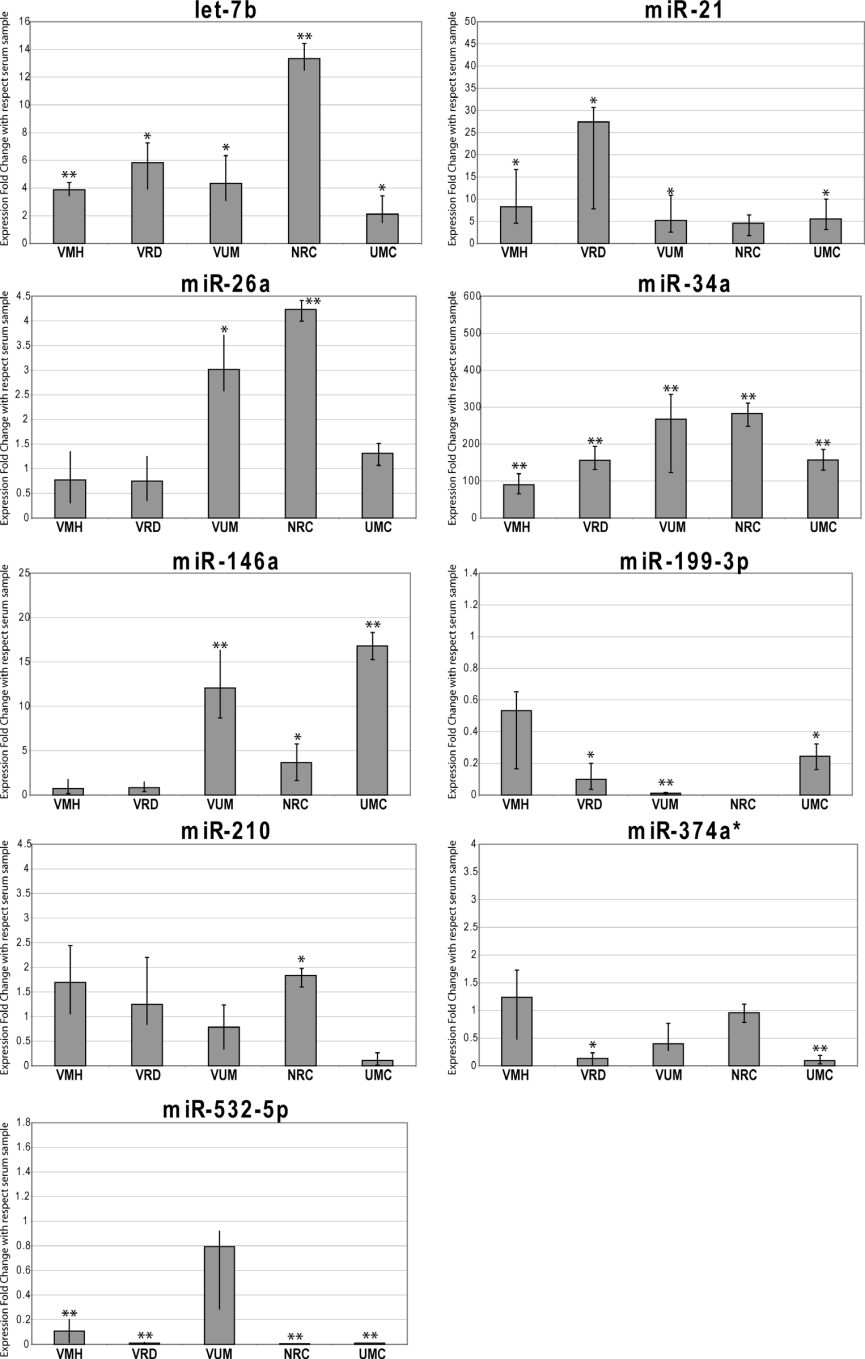
MiRNA expression in human vitreous humor. Expression levels of let-7b, miR-21, miR-26a, miR-146a, miR-199-3p, miR-210, miR-374a*, miR-532-5p in vitreous humor from patients with various ocular diseases. Values on the y-axis are reported as the mean, maximum and minimum values of the relative quantity of microRNAs (miRNAs) compared to serum. VMH=vitreous macular hole; VRD=vitreous retinal detachment; VUM=vitreous uveal melanoma; NRC=normal retinal cells; UMC=uveal melanoma cells. Wilcoxon rank-sum test, * p≤0.05; ** p≤0.01.

**Figure 2:**
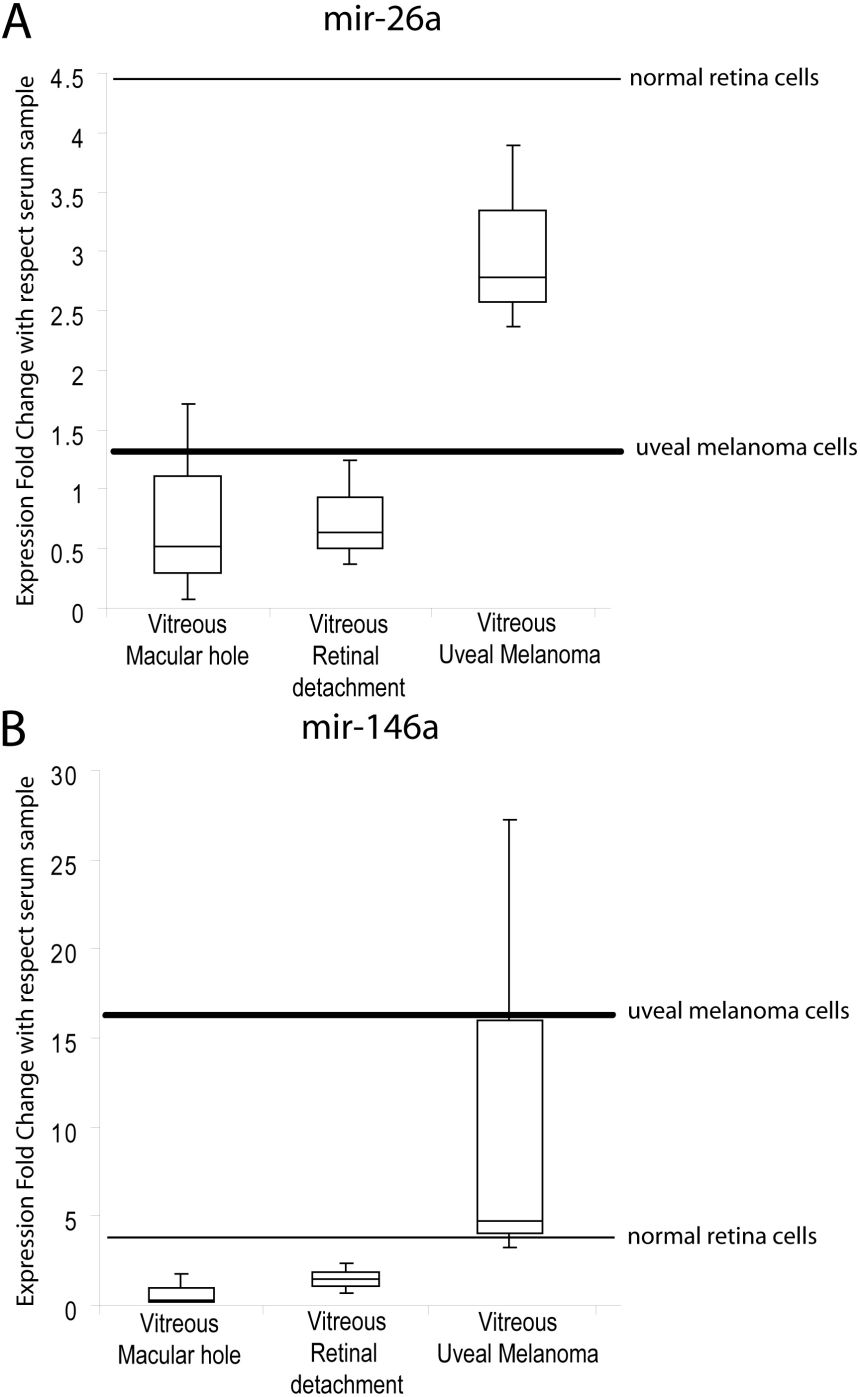
Upregulation of miR-146a and miR-26a in vitreus humor from uveal melanoma samples. Box and Whisker plot of miR-146a (**A**) and miR-26a (**B**) expression in vitreus humor (VH) from three different pathological conditions: macular hole, retinal detachment, and uveal melanoma. Values on the y-axis are reported as the relative quantity of microRNAs (miRNAs) compared to serum. Thin line: miRNA expression on normal retinal cells; thick line: miRNA expression in formalin-fixed, paraffin-embedded tissues from uveal melanoma patients. Differential expression of miRNAs in patients with uveal melanoma compared to the other pathological classes was evaluated with the Wilcoxon rank-sum test (p<0.05).

VH samples were taken from 18 patients after vitrectomy: six patients with uveal melanoma (Group A aged between 53 and 68 years, mean age 60±7), six patients with retinal detachment (Group B aged between 54 and 70 years, mean age 62±6), and six patients with macular hole (Group C aged between 52 and 68 years, mean age 60±6). By measuring the RNA concentration of the final elution from Qiagen RNA extraction procedure with a fluorometer and a spectrophotometer, we showed that human VH contains RNA (yield of 50–150 ng from 500 µl of VH). By using TaqMan Low Density Array technology, we determined the expression profile of 745 miRNAs in humor vitreous and serum from three patients affected by macular hole. We identified the presence of 94 circulating small RNAs in vitreous (including small nuclear RNA [snRNA] U6 and RNAU48) that we divided into three groups: i) highly expressed (HE), ii) normally expressed (NE), and iii) lowly expressed (LE) miRNAs (Table 1, Additional File 1). Moreover, we compared the expression levels of vitreal and serum miRNAs to verify whether these different biologic fluids had similar miRNA profiles or some expression specificity (Table 1). Interestingly, the HE group showed some miRNAs (i.e., miR-628, miR-302c, miR-639, miR-211, and miR-9) with expression fold changes higher more than 100-fold compared to serum. Furthermore, we found that some miRNAs in the NE group were greatly upregulated compared to serum: miR-452, miR-9*, miR-214, miR-184, miR-125a-3p. However, some miRNAs were strongly downregulated in vitreous HE and NE compared to serum: miR-223, miR-24, miR-484, miR-191, miR-92a, miR-30c, miR-30-5p, miR-20a, miR-150, miR-16, miR-451, and miR-93*. Taken together, these data suggest that vitreous has a specific set of circulating miRNAs that is quite different from the serum miRNA pattern. To test whether some of these vitreal miRNAs showed expression differences among different ocular diseases, we performed RT–PCR with single TaqMan miRNA assays. To validate the reproducibility of the results, the experiments were independently repeated three times. The standard deviation of threshold cycle (Ct) for each PCR real-time experiment was σ=ranged between 0.1 and 0.4. As already shown by TLDA, assayed miRNAs were present in VH, although in different quantities (Figure 1). Let-7b, miR-21, and miR-146a were the most expressed, while miR-374a* and miR-532-5p were scarcely represented in our samples. The Cts were more than 35, accordingly at the limit of TaqMan assay sensitivity and not a reliable correct quantification (Appendix 1). Overall, higher expression of miRNAs was detected in eyes with uveal melanoma compared to the two other groups. In particular, some miRNAs were significantly overexpressed compared to the value detected in the healthy donor serum (value 1 in the histograms in Figure 1). MiR-34a was highly expressed in VH from all groups, with values about 100-fold higher than serum in patients with macular hole, 150-fold in patients with in retinal detachment, 250-fold in patients with uveal melanoma; high levels were also found in normal retinal cells (300-fold) and uveal epithelioid melanoma cells (200-fold). Let-7b was expressed in normal retinal cells at higher levels than in serum (13-fold), and was increased (four- to sixfold) in all VH samples as in the uveal melanoma cells. MiR-21 was more expressed in VH from patients with retinal detachment (25-fold) and about fivefold in the VH from the other two groups, in normal retinal cells and in uveal melanoma cells (about fivefold). MiR-146a was more expressed in the VH of patients with melanoma (15-fold), uveal melanoma cells (13 folds), and normal retinal cells (fivefold), but not in other VH samples. MiR-26a was more expressed in VH from patients with melanoma (threefold) and in normal retinal cells (fourfold), but not in melanoma cells and in VH from the other two groups. In VH from patients affected by ocular melanoma, miR-146a and miR-26a were upregulated about threefold compared to the other groups (Wilcoxon signed-rank test, p value <0.05; Figure 2). MiR-146a was upregulated in the FFPE uveal melanoma samples, while miR-26a was overexpressed in normal retinal cells FFPE and uveal melanoma cells*.*

**Table 1:** MiRNAs expressed in Vitreous Humor and their comparison with circulating miRNAs in serum.

**miRNA**	**median Ct**	**Fold Change respect to serum**	**Expression group**
hsa-miR-628–5p	15.98	**704.50**	HE
hsa-miR-518f	17.23	1.20	HE
hsa-miR-302c	22.74	**622.07**	HE
hsa-miR-1274B	23.93	0.39	HE
hsa-miR-639	25.89	**109.55**	HE
hsa-miR-1260	26.80	2.69	HE
U6 snRNA	27.07	1.36	HE
hsa-miR-720	27.11	0.91	HE
hsa-miR-211	27.31	**464.94**	HE
hsa-let-7b	27.43	2.51	HE
hsa-miR-223	27.67	0.03	HE
hsa-miR-9	27.76	**688.28**	HE
hsa-miR-1825	28.21	**20.04**	NE
hsa-miR-342–3p	28.26	1.09	NE
hsa-miR-338–5P	28.94	0.0003	NE
hsa-miR-1274A	29.38	1.26	NE
hsa-miR-596	29.78	0.33	NE
hsa-let-7e	29.85	0.61	NE
hsa-miR-24	30.00	**0.05**	NE
hsa-miR-222	30.05	0.41	NE
hsa-miR-19b	30.18	**0.06**	NE
hsa-miR-452	30.57	**34.25**	NE
hsa-miR-320	30.58	**0.18**	NE
hsa-miR-17	30.77	**0.08**	NE
hsa-miR-146a	30.78	**0.12**	NE
hsa-miR-106a	30.78	**0.09**	NE
hsa-miR-204	30.80	**26.49**	NE
hsa-miR-663B	30.88	**6.07**	NE
hsa-miR-526b	30.94	**133.22**	NE
RNU48	31.11	3.57	NE
hsa-miR-671–3p	31.17	**4.80**	NE
hsa-miR-21	31.22	0.89	NE
hsa-miR-484	31.40	**0.03**	NE
hsa-miR-9*	31.41	**127.90**	NE
hsa-miR-191	31.49	**0.05**	NE
hsa-miR-34a	31.49	**92.00**	NE
hsa-miR-214	31.61	**54.70**	NE
hsa-miR-200b	31.94	**10.98**	NE
hsa-miR-184	31.99	**462.14**	NE
hsa-miR-193b	32.18	**5.06**	NE
hsa-miR-92a	32.29	**0.02**	NE
hsa-miR-200c	32.44	**9.26**	NE
hsa-miR-892b	32.44	0.36	NE
hsa-miR-100	32.57	**9.40**	NE
hsa-miR-197	32.82	0.09	NE
hsa-miR-30c	32.95	**0.03**	NE
hsa-miR-574–3p	32.97	0.50	NE
mmu-miR-374–5p	33.09	0.47	NE
hsa-miR-125a-3p	33.18	**36.21**	NE
hsa-miR-875–5p	33.24	**0.25**	NE
hsa-miR-142–3p	33.31	0.39	NE
hsa-miR-30a-5p	33.37	**0.02**	NE
hsa-miR-20a	33.38	**0.02**	NE
hsa-miR-1290	33.47	**0.34**	NE
hsa-miR-328	33.54	**0.07**	NE
hsa-miR-199a-3p	33.57	0.30	NE
hsa-let-7c	33.63	**5.09**	NE
hsa-miR-25	33.66	**0.08**	NE
hsa-miR-150	33.93	**0.01**	NE
hsa-miR-454	33.96	0.75	NE
hsa-miR-340*	33.96	0.62	NE
hsa-miR-29a	33.98	1.17	NE
hsa-miR-331	34.19	**0.07**	NE
hsa-miR-16	34.22	0.00	NE
hsa-miR-190b	34.25	**5.68**	NE
mmu-miR-451	34.30	**0.01**	NE
hsa-miR-151–3p	34.57	**0.01**	NE
hsa-miR-106b	34.91	**0.15**	NE
hsa-miR-125b	34.94	1.02	NE
hsa-miR-30e-3p	34.95	0.08	NE
hsa-miR-28–3p	34.97	0.10	NE
hsa-let-7d	34.98	0.22	NE
hsa-miR-99a	34.99	2.65	NE
hsa-miR-93*	34.99	**0.05**	NE
hsa-mir-26a	34.99	0.48	NE
hsa-miR-483–5p	35.09	0.34	NE
hsa-miR-345	35.17	**0.19**	NE
hsa-miR-210	35.20	0.90	NE
hsa-miR-223*	35.39	0.24	NE
hsa-miR-126	35.54	**0.0022**	LE
hsa-miR-374	35.66	0.30	LE
hsa-miR-19a	35.92	**0.03**	LE
hsa-miR-20b	35.96	**0.06**	LE
hsa-miR-378	35.97	0.65	LE
hsa-miR-145	35.98	**0.02**	LE
hsa-miR-146b	35.99	**0.06**	LE
hsa-miR-133a	35.99	**0.11**	LE
hsa-miR-19b-1*	36.00	1.85	LE
hsa-miR-186	36.94	**0.01**	LE
hsa-miR-195	36.95	**0.09**	LE
hsa-miR-505*	36.97	**0.13**	LE
mmu-miR-93	37.00	**0.01**	LE
hsa-miR-532–5p	37.00	**0.11**	LE
hsa-miR-374a*	37.23	0.58	LE

Highly expressed (HE), normally expressed (NE), lowly expressed. The fold change values typed in bold fonts showed statistically significant expression differences respect to serum: SAM, two-class paired test; False Discovery rate <0.15.

## Discussion

The aim of this study was to investigate the presence of miRNAs in human VH. We detected with TLDAs the presence of 92 vitreal miRNAs, some of which (miR-302c, miR-639, miR-9, miR-526b) were particularly abundant in VH but not detectable in serum. Moreover, we assayed with single TaqMan assays the expression of miRNAs that have been found in retinal cells or serum, and compared their expression in VH from different ocular pathologies to serum from healthy donors (used as the calibrator sample), normal retina cells, and epithelioid uveal melanoma cells.

Notably, only a few circulating miRNAs (94) were detectable in vitreous compared with serum. These differences were identified by comparing the expression analysis of vitreous and serum from the same individuals, suggesting that a specific set of miRNAs is secreted in the eye. The relatively small number of vitreal miRNAs and their expression specificity could suggest selected roles for these miRNAs in the eye’s vitreal chamber, for instance, the exchange of molecular signals among retina cells with each other, and/or among the few cells floating in the vitreous (i.e., phagocytes, hyalocytes of Balazs). Among the 94 miRNAs identified, snRNA U6 and RNAU48 were present. These miRNAs have a functional role in splicing within the nucleus (nRNA U6) and rRNA processing within the nucleolus (RNAU48), and are abundantly detected in many biologic fluids and exosomes. Their molecular and biologic role among circulating molecules remains unexplained. Interestingly, the most abundant vitreal miRNAs (i.e., miR-9, miR-9*, miR-125a-3p, miR-184, miR-211, miR-214, miR-302c, miR-452, miR-628, miR-639) are involved in the physiology and development of the central nervous system and the eye [32-35]. Specifically, the couple miR-9/miR-9* targets two components of the REST complex, a transcription factor that silences neuronal gene expression in non-neuronal cells [36].

Since normalizator genes are unknown in VH, canonical miRNA quantification was not possible. Accordingly, we applied the median C_t_ of all miRNAs in each sample as the normalizator and serum as the calibrator sample [30,31]. We excluded any possible source of contamination (other cells or blood) with the precise technique of sample collection and purification, as specified in the Methods section. Definitely, the source of miRNAs are the cells, but the presence of miRNAs in biologic fluids represents a critical point of debate. The most accepted hypothesis proposes that miRNAs are actively secreted in membrane-bounded-vesicles (e.g., exosomes, microvesicles) [37,38]. However, recent studies have shown that the majority of circulating miRNAs are present in plasma and serum in a non-membrane bound form, but rather protected by protein complexes (for instance, with Ago2, NPM1, or other RNA binding proteins) [39]. Moreover, the hypothesis that circulating miRNAs are released in the extracellular space from dead cells as byproducts has not been clearly ruled out [40]. Our data show for the first time the presence of miRNAs in human VH, but do not exclude any of these models. Retinal cells could physiologically exchange molecular signals with each other through miRNAs secreted into the VH [37,41]. A low metabolic exchange exists between systemic circulation and vitreous humor; therefore, fluid in the vitreous chamber could be considered stagnant. Accordingly, if blood, cells, or other byproducts of inflammation (e.g., secreted miRNAs) get into the vitreous, they could be detected there. It may be hypothesized that in the case of ocular diseases the secretory ability of retina cells could be altered, or there could be a molecular contribution from other cell types; therefore, the quality and quantity of the circulating miRNAs in VH would change, depending on the type of pathological stress applied to the retinal cells. However, circulating miRNAs in VH could be the passive result of physiologic and pathological flaking of retinal or other cells inside the ocular fluid [42]. Ideally, the human vitreous of healthy subjects should have been used as the control. In this study, this has not been done because of ethical and technical reasons; the serum of healthy subjects was used as the control.

Our data also show that some miRNAs were more represented in specific ocular conditions: MiR-26a and miR-146a were overexpressed in VH from patients affected by uveal melanomas. The biologic significance of such findings in the eye must be further investigated. For instance, miR-146a expression is increased in VH and in melanoma cells, while miR-26a is increased in VH from melanoma patients, but not in melanoma cells. This observation suggests different biologic functions of these miRNAs. Interestingly, miR-146a is also upregulated in cells from patients affected by cutaneous melanoma and in serum from patients with colorectal and lung cancer [11,43]. MiR-146a regulates extracellular matrix protein in retinal microvessels, miR-146a is involved retinal neovascularization, and the upregulation of miR-146a induces the overexpression of VEGF: interestingly, VEGF is elevated in ocular fluids of eyes with uveal melanoma [44-47]. However, upregulation of circulating miR-26a in serum has been shown to be related to prostate cancer [48]; furthermore, this miRNA is actively involved in retina cell functions, since it is a key post-transcriptional regulator of the photoreceptors’ L-type voltage-gated calcium channel α1C subunit by controlling the circadian rhythms of mRNAs in the retina [49]. Interestingly, miR-26a promotes vascular proliferation inhibiting cellular differentiation and apoptosis and inducing VEGF expression [50,51]. In addition to the biologic interest of these data, detecting specific miRNAs in VH could improve the diagnosis of several eye diseases. For instance, identifying markers for uveal melanoma could help in differentiating small uveal melanomas from benign nevi. Thus far, clinical features and ultrasonography allow a proper diagnosis, but in uncertain cases, it is necessary to perform tumor biopsy. This procedure can lead to several complications, including hemorrhage, retinal detachment, cataract, endophthalmitis, as well as tumor seeding, tumor recurrence, or extraocular spread [52]. VH aspiration also presents risks and cannot be proposed as a widely used technique. Detecting miRNAs specifically associated with ocular diseases, with the aim of a possible identification in the serum for a future quick non-invasive diagnosis, would be beneficial. In conclusion, this study successfully detected the expression of selected miRNAs in vitreous. Further studies are needed to profile the entire human set of miRNAs in the vitreous from different ocular diseases to detect those specifically associated with specific eye pathologies for diagnostic and therapeutic applications in ocular medicine.
